# The association between acute graft-versus-host disease and antimicrobial peptide expression in the gastrointestinal tract after allogeneic stem cell transplantation

**DOI:** 10.1371/journal.pone.0185265

**Published:** 2017-09-21

**Authors:** Daniela Weber, Katrin Frauenschläger, Sakhila Ghimire, Katrin Peter, Isabella Panzer, Andreas Hiergeist, Markus Weber, Daniel Kutny, Daniel Wolff, Matthias Grube, Elisabeth Huber, Peter Oefner, Andre Gessner, Thomas Hehlgans, Wolfgang Herr, Ernst Holler

**Affiliations:** 1 Department of Hematology and Oncology, Internal Medicine III, University Medical Center, Regensburg, Germany; 2 Department of Pathology, University of Regensburg, Regensburg, Germany; 3 Institute of Clinical Microbiology and Hygiene, University Medical Center, Regensburg, Germany; 4 Department of Orthopedic Surgery, University Medical Center, Regensburg, Germany; 5 Institute of Functional Genomics, University of Regensburg, Regensburg, Germany; 6 Institute of Immunology, Regensburg Center for Interventional Immunology (RCI) and University Medical Center of Regensburg, Germany; University of Kentucky, UNITED STATES

## Abstract

Intestinal microbiota disruption is associated with acute gastrointestinal (GI) Graft-versus-Host Disease (GvHD) and poor outcome after allogeneic stem cell transplantation (ASCT). Here, in a retrospective analysis of 200 patients undergoing ASCT at the Regensburg University Medical Center, we assessed the relative expression of Paneth cell antimicrobial peptides (AMPs), Human Defensins (HD) 5 and 6 and regenerating islet-derived 3α (Reg3α), in 292 human intestinal biopsies as well as Reg3α serum levels in relation to acute GI GvHD. In the absence of GI GvHD, the relative expression of Paneth cell AMPs was significantly higher in the small intestine (duodenum to ileum) than in the stomach and large intestine (cecum to rectum) for Reg3α (p≤0.001), HD5 (p≤0.002) and HD6 (p≤0.02). Acute stage 2–4 GI GvHD was associated with reduced expression of AMPs in the small intestine (p≤0.01) in comparison to stage 0–1 disease, accompanied by a decrease in Paneth cell count in case of severe acute GI GvHD (p<0.001). The opposite held true for the large intestine as we found stage 2–4 GI GvHD correlated with significantly higher expression of HD5, HD6, and Reg3α compared to mild or no acute GI GvHD (p≤0.002). Severe GI GvHD in both the lower and the upper GI tract also correlated with higher serum concentrations of Reg3α (p = 0.002). As indirect markers of intestinal microbiome diversity low levels of urinary 3-indoxyl sulfate levels were associated with severe stages of acute GI GvHD compared to mild stage or no acute GI GvHD (p = 0.05). In conclusion, acute GI GvHD correlates with intestinal expression of HD5, HD6 and Reg3α as well as Reg3α serum levels and is associated with intestinal dysbiosis.

## Introduction

The gut microbiota play a central role in the outcome of allogeneic stem cell transplantation (ASCT). [1, 2] During the course of ASCT a loss of intestinal microbiota diversity and a shift toward an enteropathogenic flora were observed first in mice models and later in humans. [3, 4] Major shifts in microbial composition were especially pronounced in individuals, who received antibiotic treatment or suffered from acute gastrointestinal (GI) Graft-versus-Host Disease (GvHD). [4] Particularly commensal bacteria, e.g. *Clostridiales*, seem to be crucial for the maintenance of immunological homeostasis and intestinal epithelial integrity and for providing anti-inflammatory effects as demonstrated in both mouse models [5, 6] and humans. [1, 7]

Paneth cells play an important role in the maintenance of a balanced gut microbiota composition and contribute to intestinal homeostasis and innate immunity. [8] Located mainly at the crypt base of the small intestine, Paneth cells, by sensing bacteria and bacterial antigens, release various antimicrobial peptides (AMPs) including enteric α–defensins, lysozyme and secretory phospholipase A2 into the small intestinal lumen. [9] They function as important regulators of microbial density, prevent microbial invasion into the crypt microenvironment and protect intestinal stem cells. [10] In humans two enteric α–defensins, human defensin (HD) 5 and 6, have been identified. [9, 10] Another important AMP secreted by Paneth cells is regenerating islet-derived 3α (Reg3α). [11] Together they exert a wide antimicrobial activity against Gram-positive and Gram-negative bacteria. Paneth cell defensins are involved in the pathogenesis of inflammatory bowel disease (IBD) such as Crohn’s disease, where their reduced expression has been linked to an inadequate Wnt ligand stimulation by defective monocytes. [12] In acute GI GVHD, the extent of Paneth cell destruction has been observed to correlate with severity of acute GvHD. [13]

In this retrospective study of patients undergoing ASCT, we analyzed relative gene expression of Paneth cell AMPs HD5, HD6 and Reg3α in 292 intestinal biopsies and determined serum levels of Reg3α in relation to acute GvHD of the GI tract.

## Patients, material and methods

### Patients

A total of 200 adult patients undergoing ASCT at the University Hospital Regensburg were included in our retrospective analysis. Inclusion criteria were hemato-oncologic disease requiring ASCT with an age above 18 years and receiving non-T cell depleted grafts. The Ethics Committee of the University Medical Center of Regensburg approved the study (02/220) and all patients participated after receipt of written informed consent. None of the transplant donors were from a vulnerable population and all donors or next of kin provided written informed consent that was freely given. Intestinal biopsies were obtained in patients with clinical suspicion of acute GI GvHD by endoscopy. In case of a second episode of an acute GI GvHD a re-endoscopy was performed including a corresponding biopsy harvesting. Beside specimens for routine microbiological and histological workup, an additional biopsy of a macroscopic suspicious mucosa (erythema, edema, erosion or ulceration) was obtained for scientific investigation. In addition, the protocol allowed inclusion of screening biopsies from asymptomatic patients early after ASCT. 72% of all biopsies were taken within the first 180 days post-transplant with a mean time of 55 days after ASCT since classic acute GI GvHD and late acute GI GvHD occur usually within the first 180 days after ASCT. [14] 28% of the biopsies were taken after day 180 with a mean of 381 days post-transplantation from patients either suffering from prolonged delayed GI GvHD or developing GI GvHD after donor lymphocyte infusion or cessation of immunosuppression because of imminent relapse.

All biopsies were stored at -20°C until analysis. For PCR analysis of HD5 and 6 and Reg3α, biopsies were classified with regard to the distribution of Paneth cells and the location of biopsy harvesting into stomach, small intestine comprising duodenum, jejunum and ileum and large intestine including biopsies from the colon and rectum. Paneth cell counts were evaluated in histological sections of intestinal biopsies. To further investigate the association between Reg3α serum levels and acute GI GvHD we performed Reg3α serum measurements within seven days around the day of biopsy harvesting. Urinary 3-Indoxyl sulfate (3-IS) levels analyzed previously and obtained within 7 days of endoscopy for inpatients and 14 days for outpatients were used for assessment of microbiome diversity at the time of biopsy. Furthermore, 3-IS measurements within the first 4 weeks after ASCT were available for all samples with Paneth cell measurements. Patients’ characteristics are shown in Table 1.

**Table 1:** Summary of patient characteristics.

**Median age in yrs (range)**	51.6 (17.1–70.7)
**Diagnosis** Acute leukemia Lymphatic neoplasia Myelodysplastic syndrome (MDS) Myeloproliferative syndrome (MPN) Aplastic anemia	114 (57.0%) 52 (26.0%) 21 (10.5%) 9 (4.5%) 4 (2.0%)
**Stage of underlying disease** Early / intermediate Advanced	110 (55.0%) 90 (45.0%)
**Donor** Sibling Unrelated donor	57 (28.5%) 143 (71.5%)
**Conditioning** RIC Standard	172 (86.0%) 28 (14.0%)

**Fig 1:**
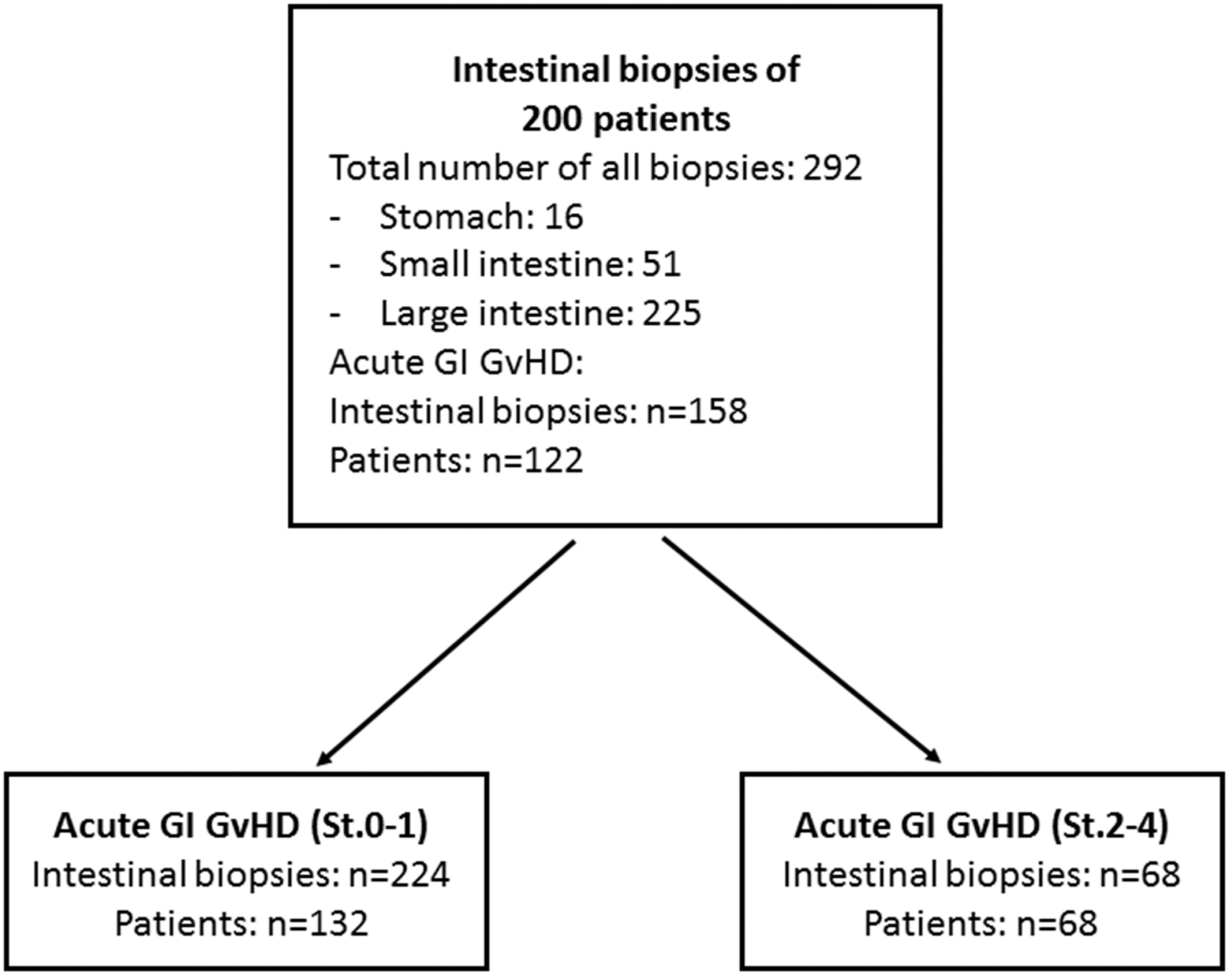
Flow chart describing the study design as well as biopsy numbers in all groups.

GvHD in the upper and lower GI tract was staged according to the Glucksberg grading system. [15] In case of initial clinical signs of acute GI GvHD like diarrhea, abdominal cramping, nausea or vomiting a routine endoscopy was performed within 48 to 72 hours after clinical onset of aGvHD symptoms for further clarification of the clinical suspicion. A total of 122 (61.0%) of the patients developed acute GI GvHD of the upper or lower GI tract. Out of this subgroup 68 (34.0%) patients showed severe stages (St. 2–4) of acute GI GvHD (Fig 1).

### Analysis of HD5 and 6 and Reg3α

Quantitative real-time PCR (qPCR) was performed on a total of 292 intestinal biopsies for HD5, HD6 and Reg3α as described earlier.(16) RNAs were stored at -80 degrees to avoid long term degradation. Following RNA extraction, the quality of RNA was determined by bio-analyzer and RIN score was between 7 and 9.

18S ribosomal RNA was used as a reference gene. Gene of interest was normalized to 18S rRNA thus relative expression was evaluated. Following gene-specific primers were used: DEFA5, forward: 5’-AGA-CAA-CCA-GGA-CCT-TGC-TAT-CTC, reverse: 5’-GGT-TCG-GCA-ATA-GCA-GGT-GG; DEFA6, forward: 5’-GAC-CAG-GAC-TTT-GCC-GTC-TC, reverse: 5’-CAA-GTG-AAA-GCC-CTT-GTT-GAG-CC; REG3A, forward: 5’-ATC-CGC-TGT-CCC-AAA-GGC-TC, reverse: 5’-AGC-ACA-GAC-ACC-AGG-TTT-CCA-G; 18S, forward: 5’-ACC-GAT-TGG-ATG-GTT-TAG-TGA-G, reverse: 5’-CCT-ACG-GAA-ACC-TTG-TTA-CGA-C. [16]

### Analysis of Reg3α in the serum

In 126 patients giving 175 biopsies Reg3alpha serum levels were analyzed within seven days around day of biopsy harvesting in duplicate by using indirect ELISA as previously described. [11] Mean was calculated out of duplicates. Standard curve was drawn for each experiment where the optical density of sample always fell within the standard curve. The distribution of patients with severe and non-severe stages of acute GI GvHD was comparable to the distribution in the whole study group.

### Analysis of urinary 3-indoxyl sulfate (3-IS) levels

In a total of 63 patients 3-IS levels were analyzed at the time point of biopsy harvesting as recently described. [7]

### Histopathology

The number of Paneth cells was determined with a Zeiss Axioskop 40 microscope in hematoxylin and eosin (HE) stained sections of intestinal biopsies of the duodenum (n = 51). Additionally the number of Paneth cells was assessed in a randomly chosen cohort of 22 patients with colon biopsies (n = 34). Paneth cells were counted in at least 3 high-power fields (hpf) in the area of each biopsy showing the largest number of Paneth cells. The counts from each hpf were then averaged to give the number of Paneth cells per hpf. (13) An hpf was defined as the visible area in a 400 x magnification (0.307 mm^2^).

All slides were reviewed by two pathologists (one fellow and one attending) independently, not blinded to clinical data. In case of disagreement, the attending overruled the fellow’s results.

### Bioinformatics and data analysis

Continuous data are presented descriptively as median (range). Group comparisons were performed by two-sided Mann-Whitney-U-Tests or correspondingly Kruskal-Wallis tests due to non-normal data distribution. Statistical analyses were performed using IBM SPSS Statistics 22 (SPSS Inc, Chicago, IL, USA).

## Results

### Expression of Paneth cell AMPs differs between stomach, small intestine and large intestine

**Fig 2:**
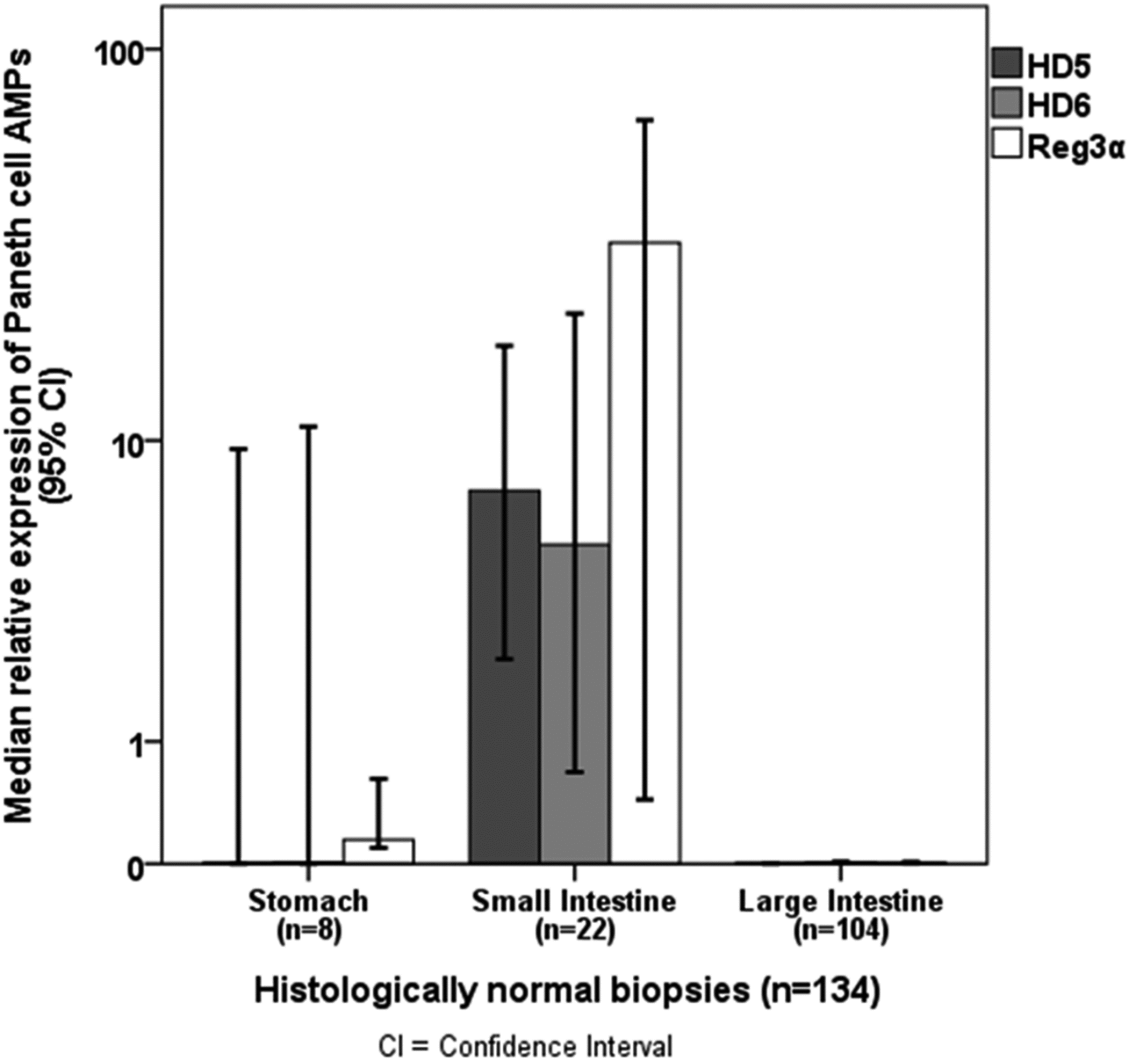
Distribution of Paneth cell AMP expression in relation to the location of biopsy harvesting in the gastrointestinal tract. The relative expression of the Paneth cell AMPs HD5, HD6 and Reg3α was higher in the small intestine (n = 22) than in the stomach (n = 8) and large intestine (n = 104) in histologically normal biopsies (n = 134, p<0.02, Mann-Whitney-U-Test).

For all three Paneth cell AMPs (Reg3α, HD5 und HD6), we found significant differences in relative expression between stomach, small intestine and large intestine according to the physiological distribution of Paneth cells in the GI tract. The relative expression of HD5 and HD6 was higher in histologically normal biopsies (n = 134) of the small intestine than the stomach and large intestine with 7.3 (8.7x10^-5^ to 5.5x10^1^) vs. 2.7x10^-3^ (8.1x10^-5^ to 9.5, p = 0.002) and 1.2x10^-3^ (0 to 2.5x10^-1^, p<0.001) for HD5 and 5.1 (1.3x10^-4^ to 8.6x10^1^) vs. 9.4x10^-3^ (5.2x10^-4^ to 1.1x10^1^, p = 0.02) and 9.5x10^-3^ (1.3x10^-4^ to 8.6x10^1^, p<0.001) for HD6. Interestingly, we found also significantly higher Reg3α expression in the small intestine (3.3x10^1^, 5.1x10^-4^ to 2.6x10^2^) than in the stomach (1.5x10^-1^, 1.9x10^-2^ to 8.3x10^1^, p = 0.001) or large intestine (3.1x10^-3^, 3.1x10^-5^ to 9.2x10^1^, p<0.001, Fig 2).

### Levels of Paneth cell AMPs correlate with the severity of acute GI GvHD

**Fig 3:**
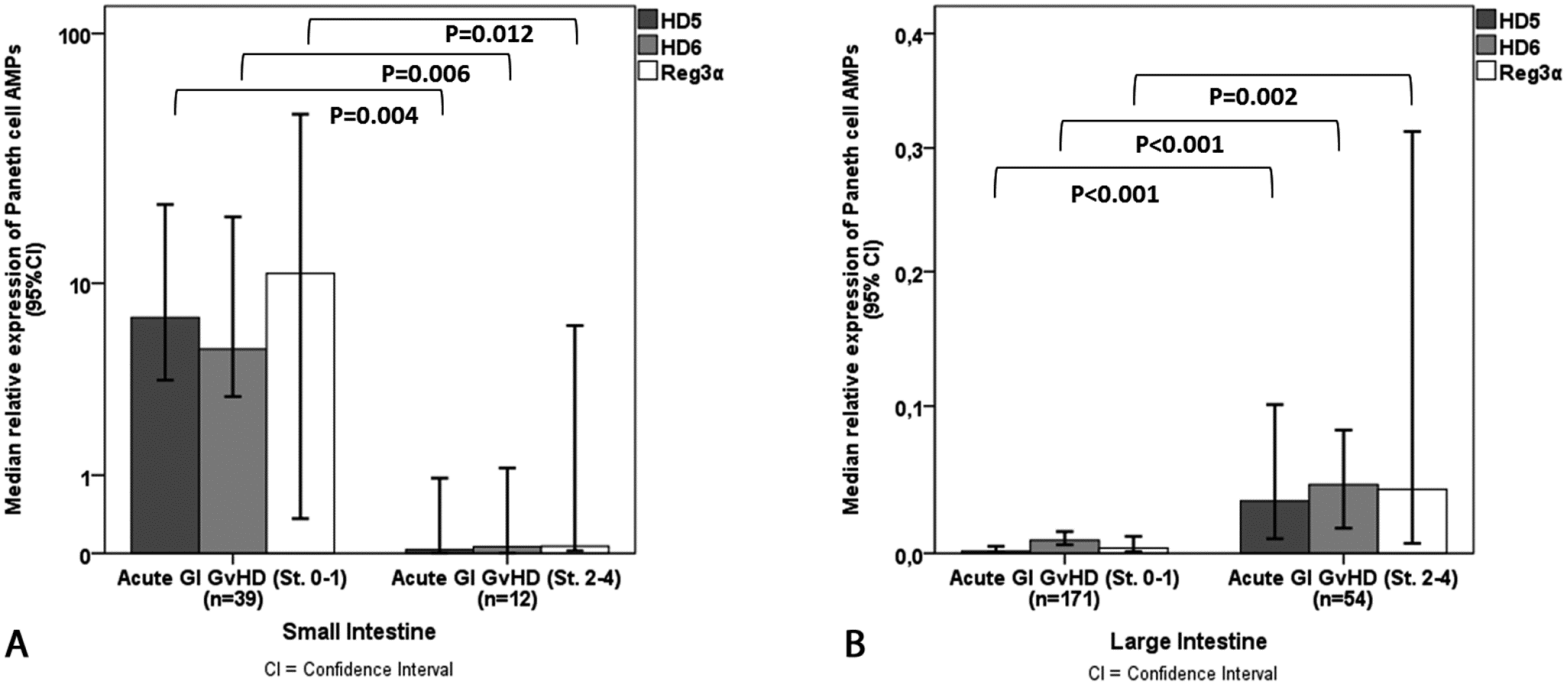
Relative expression of Paneth cell AMPs correlates with severity of acute GI GvHD. In the small intestine the relative expression of HD5 (p = 0.004), HD6 (p = 0.006) and Reg3α (p = 0.012) was significantly decreased (Mann-Whitney-U-Test) in case of severe acute GvHD (stage 2–4) (3A). Patients with acute intestinal GvHD stages 2–4 showed an increase in expression of HD5 (p<0.001), HD6 (p<0.001) and Reg3α (p = 0.002) in the large intestine (Mann-Whitney-U-Test) (3B).

Next, we investigated the impact of acute GI GvHD on the relative expression of Paneth cell AMPs in acute GI GvHD. Severe acute GI GvHD (stage 2–4) was associated with a significant decrease in AMPs in the small intestine compared to mild stage or no GI GvHD (stage 0–1) (Fig 3A). We found a drop of HD5 expression in patients with severe acute GI GvHD from 7.1 (8.7x10^-5^ to 8.0x10^1^) to 3.2x10^-2^ (1.8x10^-4^ to 1.6x10^1^, p = 0.004), for HD6 from 5.1 (1.3x10^-4^ to 2.2x10^2^) to 6.0x10^-2^ (3.6x10^-4^ to 3.0x10^1^, p = 0.006) and for Reg3α from 1.1x10^1^ (5.1x10^-4^ to 3.3x10^2^) to 6.6x10^-2^ (1.2x10^-4^ to 5.2x10^1^, p = 0.012), respectively. Reduced expression of AMPs was accompanied by a decrease in Paneth cell count from 18.6 (0–79.0) to 2.8 (0–42.6, p<0.001) in case of severe GI GvHD. In the large intestine, in contrast, stage 2–4 GI GvHD correlated with significantly higher expression of HD5, HD6, and Reg3α (Fig 3B), as we observed an increase of HD5 in patients with severe stages of GI GvHD from 1.4x10^-3^ (0 to 2.1) to 3.4x10^-2^ (9.1x10^-5^ to 3.7x10^1^, p<0.001), for HD6 from 8.5x10^-3^ (1.9x10^-4^ to 2.0) to 4.5x10^-2^ (1.1x10^-3^ to 3.4x10^1^, p<0.001) and for Reg3α from 3.3x10^-3^ (3.1x10^-5^ to 2.3x10^1^) to 4.2x10^-2^ (1.0x10^-4^ to 5.2x10^1^, p = 0.002), respectively. A metaplasia of Paneth cells in the large bowel was not found in the presence of severe acute GI GvHD as we observed Paneth cells in 12 of 34 colon biopsies. Accordingly, mean Paneth cell count in the large intestine was measured with 0.3 (0–37.4) in patients with no/mild GI GvHD and with 0.3 (0–26.0, p = ns) in patients with GI GvHD stage 2–4, respectively. Severe GI GvHD in both the lower and the upper GI tract also correlated with higher serum concentrations of Reg3α (161.5 ng/ml, 8.5–1744.0 ng/ml) as compared to mild GI GvHD (63.2 ng/ml, 4.5–2296 ng/ml, p = 0.002). Due to the low number of stomach biopsies an analysis of GvHD changes in AMP secretion was not possible for this subgroup of patients.

### Severe acute GI GvHD and Paneth cell number correlate with loss of microbiome diversity

As indirect markers of intestinal microbiome diversity, urinary 3-IS levels were measured within 14 days of biopsy harvesting. Severe stages of acute GI GvHD were associated with lower levels of 3-IS with 0.001 μmol/mmol creatinine (0 to 0.3 μmol/mmol creatinine) compared to 0.06 μmol/mmol creatinine (0 to 99.0 μmol/mmol creatinine) in patients with mild stage or no acute GI GvHD (p = 0.05). The correlation of Paneth cell number and 3-IS showed a trend (data not shown). Instead, researching into early microbiome disruption within the first 4 weeks after ASCT, a higher number of Paneth cells with 25.0 (0–79.0) was found in patients with early high 3-IS levels (> median) compared to 13.0 (0–33.0) in patients with early low 3-IS levels (p = 0.01).

## Discussion

The composition of intestinal microbiota seems to play a key role in the pathophysiology of acute GI GvHD and has been reported to influence the outcome of ASCT recipients. [1, 2] Paneth cells contribute essentially to the maintenance of a highly diverse intestinal microbiome and intestinal homeostasis by the production of AMPs. [17] Physiologically, Paneth cells are mainly located in the small intestine and in smaller numbers in the proximal large intestine as well. In response to microbial antigens, Paneth cells secrete AMPs into the lumen of the crypt and, thus, prevent microbial invasion into the crypt environment. [8, 9] According to the physiological distribution of Paneth cells in the GI tract, we observed a significantly higher relative expression of Paneth cell AMPs HD5, HD6 and Reg3α in mucosal biopsies of the small intestine including duodenum, jejunum and ileum compared to the large intestine comprising colon and rectum.

AMPs are small cationic peptide antibiotics with bactericidal activity against pathogenic bacteria, fungi and several viruses. [17] However, they provide only minimal or no antimicrobial activity against commensal bacteria and, therefore, regulate the composition of the intestinal microbiota. [8, 10] Nakamura et al reported, that mice lacking matrix metalloprotease 7, responsible for processing and activating mouse cryptidins, which fall in the category of defensins, showed a greater number of surviving enteropathogenic bacteria and a higher susceptibility to systemic disease after challenging orally with E. coli or Salmonella typhimurium. These results demonstrate that Paneth cells are involved in mammalian host defense by the secretion of cryptidins in response to various stimuli like carbamylcholine, Gram-positive and Gram-negative bacteria, lipopolysaccharide and muramyl dipeptide. [8] Masuda et al demonstrated also in mouse models, that cryptidin 4, which is the most abundant mouse Paneth cell alpha-defensine, shows bactericidal activity against pathogenic bacteria but no activity against 8 out of 12 commensal bacterial species, including Bifidobacterium bifidum and Lactobacillus casei. [18]

Several genetic defects in Paneth cells have been reported in patients with Crohn´s disease resulting in a weakened mucosal antimicrobial defense and alterations of the intestinal microbiota. These findings suggest a key role of these cells in the pathophysiology of IBD. Interestingly, NOD2/CARD15 polymorphisms, which have also been described as risk factors of GvHD by our group, are among the most important genetic defects in IBD. [19–21] Further, Paneth cells are also targeted by acute intestinal GvHD resulting in an impaired secretion of AMPs. Recently, Eriguchi and colleagues described a reduced fecal expression of α-defensin cryptidin 1 and a consequent loss of intestinal microbiota diversity in mice developing acute GI GvHD. [22] Our analyses of Paneth cell AMPs in intestinal biopsies of patients with acute GI GvHD showed an opposing correlation of GvHD and relative AMP expression in the small intestine compared to the large intestine. The presence of severe acute GI GvHD was associated with a significantly reduced expression of Paneth cell AMPs in the small intestine. This may reflect Paneth cell destruction by acute GI GvHD as reported by Levine et al. in 2013. [13] In the large intestine, on the other hand, we found a significant increase in the expression of HD5, HD6, and Reg3α in the presence of severe acute GI GvHD, although to a much lower extent than seen in the upper GI biopsies. A suspected metaplasia of Paneth cells in the large intestine in the presence of GI GvHD was not supported by our data. In contrast, Paneth cell metaplasia was previously described in patients with IBD as well as in the regenerating bowel of neonates after necrotizing enterocolitis. [19] However, not only Paneth cells are able to produce AMPs, also intestinal epithelial cells are able to secrete AMPs into the intestinal lumen in order to limit intestinal inflammation and to restore intestinal homeostasis, [23] which might explain inflammatory induction in the large intestine during GvHD as found in our data. However, in fact it cannot be ruled out that other factors, e.g. SNPs, prior therapies damaging the gut, conditioning and GVHD prophylaxis such as MMF caused alterations in the microbiota contributing to the development or worsening of acute GI GVHD.

In 2011, Ferrara et al. described Reg3α as biomarker in the serum of patients with acute intestinal GvHD. The mucosal damage due to intestinal inflammation leads to microscopic breaches in the mucosal epithelial barrier permitting a transmission of Reg3α from the mucus into the systemic circulation. [11] The presence of severe acute GI GvHD was not only associated with significant changes of the Reg3α expression in intestinal mucosa, we also measured significantly higher Reg3α serum levels in patients with severe GvHD stages confirming the data described by Levine et colleagues. [13]

In the literature, acute GI GvHD was found to be associated with distinct disruptions of the intestinal microbiota diversity. [4] One mechanism contributing to altered microbiota composition seems to be the destruction of Paneth cells by intestinal inflammatory procedures induced by acute GI GvHD. [13] Resulting in a reduction of AMP expression the maintenance of intestinal bacterial homeostasis is impaired and a shift toward an enteropathogeneic flora occurs. [24] Beside distinct changes of AMP expression we also found lower urinary 3-IS levels at the time of biopsy harvesting in patients with severe stages of acute GvHD indicating a loss of microbiota diversity. Similar results were found by Bevin et al. showing a reduced expression of HD5 and HD6 resulting in alterations of the composition of commensal microbiota as a fundamental feature of the pathogenesis of ileal Crohn's disease. [20]

A further hint, that intestinal microbiota composition strongly correlates with the regulation of AMPs was found by Li et al. in mice. After initiation of cecal dysbiosis by antibiotic treatment with ceftriaxone a significant overproduction of defensins in the ileum and colon could be observed. However, a recovery of intestinal microbiota by fecal microbiota transplantation led to quick normalization of defensin levels. [25]

The correlation of Paneth cell loss with GvHD but also early suppression of 3-IS levels raises the question concerning the causal relationship of the observed associations between GvHD and loss of AMPs. On one hand, Paneth cell loss by GvHD might induce loss of AMPs and subsequent suppression of commensal bacteria as indicated by 3-IS levels. The strong impact of early 3-IS levels however also raises the possibility that early loss of commensals (induced by cytotoxic conditioning, SNPs of innate immunity, broad spectrum antibiotics) facilitates Paneth cell destruction (e.g. via loss of epithelial protection by IL22 [26] and loss of AMPs which further enhances GvHD and a vicious circle of microbiota damage. Careful extended and sequential analyses of microbiota changes in relation to AMP expression and GvHD are needed to clarify the interactions although both mechanisms seem to contribute based on experimental data.

In conclusion, our data suggest an association between acute GI GvHD, Paneth cells and their expression of AMPs HD5, HD6 and Reg3α in intestinal biopsies and a consecutive release of Reg3α in the blood stream. The low concentrations of 3-IS levels reflecting a disruption of the intestinal microbiota diversity provide an indication of the relevance of AMP shifts in patients undergoing ASCT.
